# Mitochondrial DNA haplogroups and short-term neurological outcomes of ischemic stroke

**DOI:** 10.1038/srep09864

**Published:** 2015-05-20

**Authors:** Biyang Cai, Zhizhong Zhang, Keting Liu, Wenping Fan, Yumeng Zhang, Xia Xie, Minhui Dai, Liping Cao, Wen Bai, Juan Du, Qiliang Dai, Shuyu Zhou, Hao Zhang, Wusheng Zhu, Minmin Ma, Wenhua Liu, Xinfeng Liu, Gelin Xu

**Affiliations:** 1 Department of Neurology, Jinling Hospital, Medical School of Nanjing University, Nanjing 210002, Jiangsu, China; 2 Department of Neurology, Jinling Hospital, Southern Medical University, Nanjing 210002, Jiangsu, China; 3 Department of Neurology, The First People’s Hospital of Changzhou, Changzhou 213003, Jiangsu, China; 4 Department of Neurology, Yantai Yuhuangding Hospital, Yantai 264000, Shandong, China; 5 Department of Neurology, Jinling Hospital, Second Military Medical University, Nanjing 210002, Jiangsu, China; 6 Department of Neurology, The First People's Hospital of Hangzhou, Nanjing Medical University, Hangzhou 310006, Zhejiang, China

## Abstract

Stroke is one of the leading causes of death and long-term disability worldwide. Mitochondrial DNA (mtDNA) is a potential contributor for the sex differences of ischemic stroke heritability. Although mtDNA haplogroups were associated with stroke onset, their impacts on stroke outcomes remain unclear. This study aimed to evaluate the impacts of mtDNA haplogroups on short-term outcomes of neurological functions in patients with ischemic stroke. A total of 303 patients were included, and their clinical data and mtDNA sequences were analyzed. Based on the changes between baseline and 14-day follow-up stroke severity, our results showed that haplogroup N9 was an independent protective factor against neurological worsening in acute ischemic stroke patients. These findings supported that mtDNA variants play a role in post-stroke neurological recovery, thus providing evidences for future pharmacological intervention in mitochondrial function.

Stroke is one of the most common cause of death and disability globally, and it had been the leading cause of mortality and disability in China^1^,^2^. Genetic factors should play a role in development of stroke because major risk factors could not fully account for the risk of stroke^3^. Supporting this theory, a number of single nucleotide polymorphisms (SNP) which may influence the risk of stroke had been identified. However, only a small portion of genetic risk of stroke has been illustrated to date. One possible reason lies in that most genetic studies only focused on autosomal DNA, and presume equal possibility of heritability from two parents. But genetic epidemiological studies observed that heritability of ischemic stroke in women is greater than that in men^4^,^5^, which means that stroke is more prone to be inherited from mother than from father. This phenomena cannot be explained by our current knowledge of autosomal susceptibility loci. To bridge this knowledge gap, study focusing on relation between non-autosomal DNA variants and stroke is warranted. With a maternal inheritance pattern, mitochondrial DNA (mtDNA) is increasingly regarded as a genetic contributor for the sex differences of ischemic stroke heritability^6^,^7^. Mitochondrial DNA codes 13 oxidative phosphorylation (OXPHOS) subunits, which are key components of oxidative respiratory chain^8^. Mutations in mtDNA can affect OXPHOS function, and subsequently result in related neuropathy^9^, such as mitochondrial encephalomyopathy, lactic acidosis, and stroke-like episodes syndrome (MELAS)^10^. MtDNA haplogroups, defined as groups of specific haplotypes of mitochondrial variants, are also determinants of the efficiency of OXPHOS^11^.

Most human cells obtain essential energy for maintaining normal functions and surviving environmental challenges via OXPHOS pathway. Given the high energy requirement, this pathway may be important for brain cells. For ischemic stroke, the efficiency of OXPHOS may be crucial for the survival of neural cells endangered ischemia^12^. In addition, mtDNA is able to mediate cellular bioenergetics and expression levels of nuclear genes in the inflammation and apoptosis pathways^13^, which are important for response to ischemic damage^12^,^14^,^15^. The pro-inflammatory/anti-inflammatory SNPs, which are able to influence inflammation, may also play important roles in stroke development^16^. Therefore, we hypothesize that mtDNA haplogroups may influence the functional outcome of ischemic stroke. We investigated the impacts of mtDNA haplogroups on 14-day outcomes of neurological functions in a cohort of Han Chinese patients with acute ischemic stroke. Short-term outcomes of acute ischemic stroke are influenced by predictors such as blood pressure, serum cholesterol levels, and serum glucose levels^17^,^18^. Use of intravenous (IV) thrombolysis, antihypertensive drugs, and antiplatelet drugs may also play a role in prognosis of acute ischemic stroke^18^,^19^. The effects of these predictors were also evaluated in this study.

## Results

In this cohort recruitment, 303 patients were enrolled and analyzed based on criteria of inclusion and exclusion. According to the changes of National Institutes of Health Stroke Scale (NIHSS) scores between baseline and 14-day follow-up, the neurological functions improved (NIHSS score decreased by ≥ 2 points) in 162 (53.5%); stabilized (NIHSS score unchanged or deceased by 1 point) in 103 (34.0%); and worsened (NIHSS score increased by ≥ 1 point) in 38 (12.5%) patients. Demographic and clinical characteristics are shown in Table 1. Clinical characteristics including prevalence of hypertension (*P* < 0.001) and prevalence of diabetes mellitus (DM, *P* = 0.006) among three neurological outcome groups showed significant differences. Ratios of using antihypertensive drugs (*P* = 0.023), ratios of using glucose-lowering drugs (*P* = 0.022) and ratios of using statins (*P* = 0.019) also differed among three outcome groups. The 14-day modified Rankin Scale (mRS) scores were significant different among three neurological outcome groups (Kruskal-Wallis *P* < 0.001); these two assessments of stroke outcomes, mRS and three levels of NIHSS score change, showed a significant correlation (Spearman *r* = 0.388, *P* < 0.001).

### Characteristics of mtDNA Haplogroups

**Figure 1:**
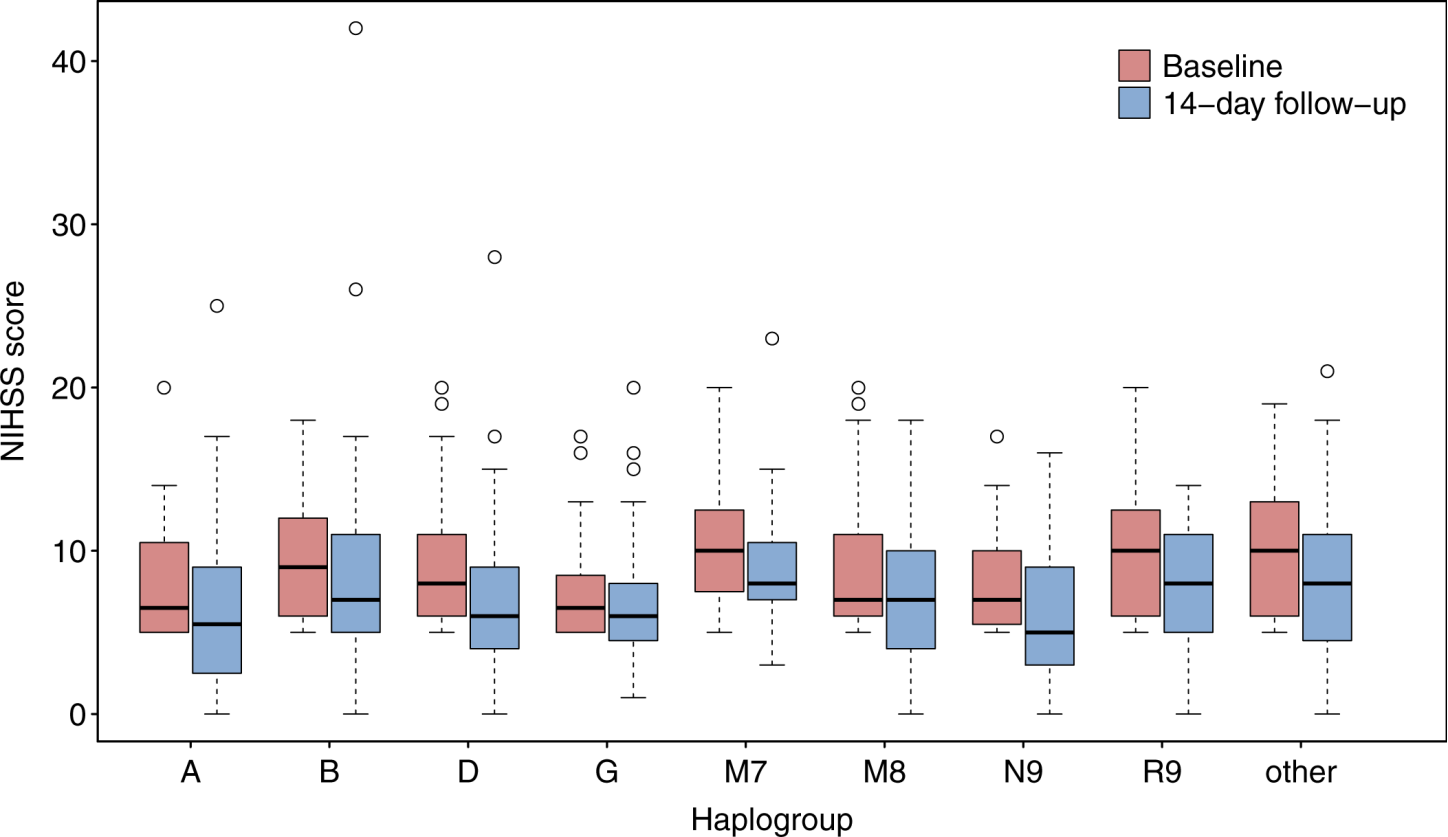
Box plot of baseline and 14-day follow-up NIHSS scores for mtDNA haplogroups.

According to mtDNA sequencing and haplogrouping results, 252 (82.5%) patients were assigned into 8 major haplogroups: A (6.6%), B (13.5%), D (21.1%), G (5.3%), M7 (8.3%), M8 (8.9%), N9 (6.3%) and R9 (13.2%). The other 51 (16.8%) patients of the other minor haplogroups were totally classified as “other” for data analyses. Detailed haplogroups and their frequencies are shown in **Supplementary Table S1** online. Baseline NIHSS scores were different among mtDNA haplogroups (*P* = 0.049); however, detailed post-hoc comparisons of haplogroups did not detect significant differences. The 14-day follow-up NIHSS scores (*P* = 0.227) and mRS scores (*P* = 0.110) did not differed among haplogroups. And demographic and clinical characteristics for mtDNA haplogroups are shown in **Supplementary Table S2** online. Both baseline and 14-day follow-up NIHSS scores of mtDNA haplogroups were shown in Fig. 1.

### MtDNA Haplogroup and Outcomes of Neurological Functions

**Figure 2:**
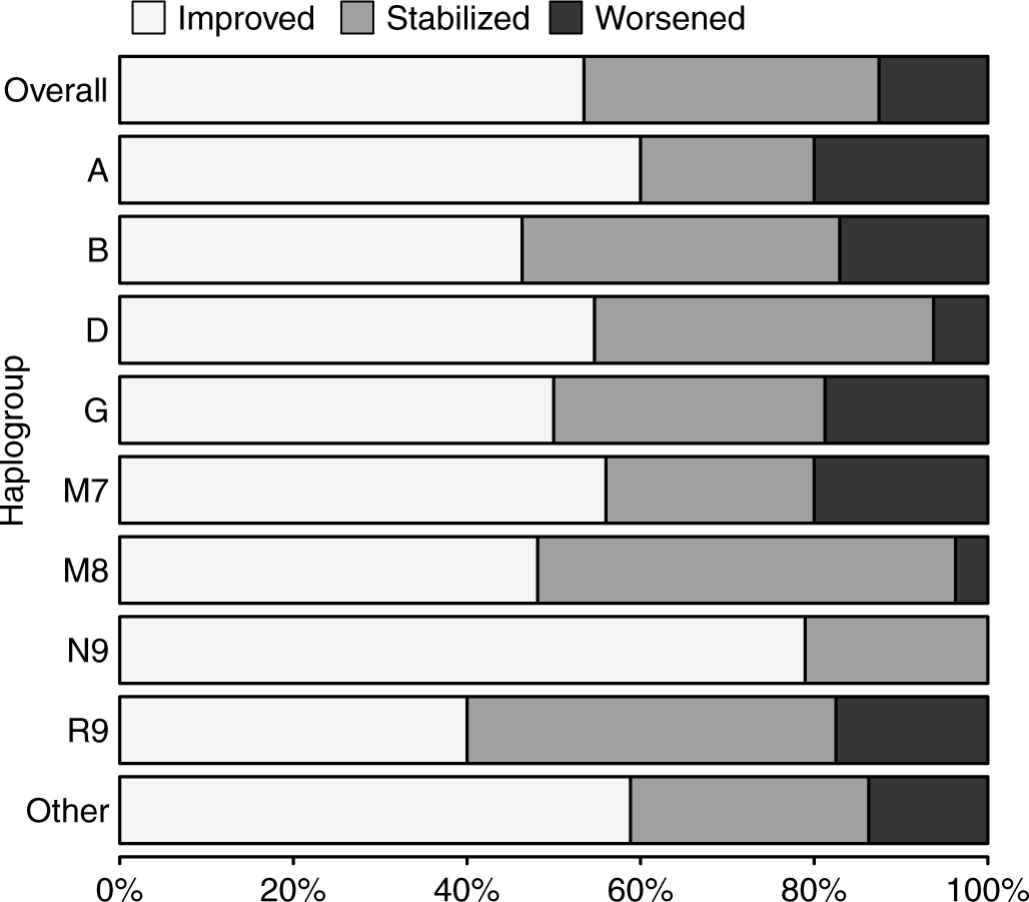
Proportions of neurological outcomes for mitochondrial DNA haplogroups. According to 14-day follow-up NIHSS score compared with baseline, the outcomes of neurological functions were classified as improved (NIHSS score decreased by ≥ 2 points), stabilized (NIHSS score unchanged or deceased by 1 point) and worsened (NIHSS score increased by ≥ 1 point).

Since differences among three outcome groups were observed in hypertension, DM, using antihypertensive drugs, using glucose-lowering drugs and using statins, these confounders were included as covariates in the ordinal regression model. The odd ratios were also adjusted for age and TOAST subtypes, which are acknowledged as important predictors for stroke outcome. After adjusted for age, hypertension, DM, TOAST subtypes, antihypertensive treatment, glucose-lowering treatment and statin treatment, haplogroup N9 was associated with a lower ratio of worse neurological outcomes (odd ratios (OR) = 0.39, 95% confidence interval (CI) = 0.20-0.75, *P* = 0.005, Bonferroni corrected *P* = 0.039). Effects of mtDNA haplogroups on outcomes of neurological functions are shown in Table 2. Proportions of neurological outcomes for mtDNA haplogroups are shown in Fig. 2.

In order to evaluate the role of treatments in stroke outcome, another regression model that included all treatments variables was also analyzed. Compared to the primary model, variables such as IV thrombolysis, antiplatelet drugs, anticoagulants, current smoking status and current alcohol drinking status was also included as covariates. In this model, the effects of these drugs were not observed (IV thrombolysis, *P* = 0.103; antihypertensive drugs, *P* = 0.537; glucose-lowering drugs, *P* = 0.255; antiplatelet drugs, *P* = 0.629; anticoagulants, *P* = 0.536). And the effect of haplogroup N9 was also significant (OR = 0.38, 95% CI = 0.20–0.74, *P* = 0.004).

### MtDNA Haplogroup and Short-term Clinical Outcome

Based on 14-day mRS, 102 (33.7%) patients had a good clinical outcome (mRS ≤ 2), and 201 (66.3%) had a poor outcome (mRS > 2). Age, TOAST subtypes and baseline NIHSS score were included as covariates in the multivariate logistic regression model. No differences concerning frequency distributions of haplogroups were observed between patients with good and poor clinical outcomes. Demographic and clinical characteristics between two clinical outcome groups are shown in **Supplementary Table S3** online. Results of associations between mitochondrial haplogroups and clinical outcomes are shown in **Supplementary Table S4** online. Proportions of 14-day mRS scores for mtDNA haplogroups are shown in **Supplementary Fig. 1** online.

### Effects of haplogroups in Sex-stratified analyses

Considering sex might be a potential confounder, the effects of haplogroups were analyzed in male and female patients separately. Haplogroup N9 was a protective factor against neurological worsening in male patients (OR = 0.45, 95% CI = 0.23-0.88, *P* = 0.019). There were only 2 female patients with N9 haplotype. Both of them had improved neurological outcomes. However, parallel lines assumption for the ordinal regression model was not met (*P* < 0.001). Thus, the effect of haplogroup N9 in female patients could not be calculated. Overall, we did not observe different effects of haplogroup N9 between male and female patients. For male patients, associations with outcomes of neurological functions between haplogroup A (OR = 0.37, 95% CI = 0.15-0.90, *P* = 0.028) and haplogroup B (OR = 1.75, 95% CI = 1.11-2.75, *P* = 0.016) were also revealed. However, the two associations of haplogroup A (adjusted *P* = 0.224) and haplogroup B (adjusted *P* = 0.128) were nonsignificant after Bonferroni correction. Haplogroup B was associated with increased risk of poor clinical outcome (OR = 3.02, 95% CI = 1.03-8.89, *P* = 0.045, Bonferroni corrected *P* = 0.357) in male patients; haplogroup A (OR = 11.60, 95% CI = 1.26-107.25, *P* = 0.031, Bonferroni corrected *P* = 0.246); the results of Bonferroni corrections indicated that these two associations may be false positives caused by multiple comparisons.

## Discussion

In this study, mitochondrial DNA haplogroup N9 was associated with better short-term outcomes of neurological functions in patients with acute ischemic stroke. These results evidenced that mitochondrial DNA also influence outcome of ischemic stroke.

As a major sub-haplogroup of N9, haplogroup N9a has been suggested as a protective factor for atherothrombotic cerebral infarction via reducing the risk of DM in Japanese males^20^,^21^. But the mechanism concerning how N9 influenced neurological recovery remains undetermined. Several ischemia-related pathways, such as OXPHOS, inflammation and apoptosis, may^13^,^22^,^23^ Subsequently, mtDNA haplogroups may determine the ratios between severely ischemic core tissues and ischemic penumbra, and influence ischemic-reperfusion cell death^12^,^24^,^25^. Neural cells with favorable mitochondrial haplogroups may have higher chances to survive the ischemia before the restoration of blood flow by collateral circulation or recanalization of the blocked artery. Theoretically, patients with advantageous mtDNA haplogroups should have smaller infarct volumes and better neurological outcomes. The results of this study provided indirect evidences to support this hypothesis.

In recent studies, mtDNA haplogroup U, H1 and K were associated with onset of stroke in European population^26^,^27^,^28^,^29^. Although these associations failed to be replicated in other two studies^30^,^31^, the accumulative effect of mitochondrial SNPs were found to be associated with ischemic stroke^31^. For associations with outcomes of stroke, haplogroup K was found unrelated to 6-month mRS^29^. Patients with macro-haplogroup R0 was also found to have a greater change of 1-month NIHSS score^32^. However, these Caucasian population-based study did not involve Asian haplogroups because of their low frequencies. Similarly, these Caucasian haplogroups present rarely in Asian population, and therefore their effects could not be evaluated in this study. Mitochondrial haplogroup D4b was also reported as a protective factor against ischemic stroke in Han Chinese population^33^. But the frequency of haplogroup D4b is very low in patients enrolled in this study; its association was not tested. A study in Japanese population found that mtDNA haplogroup A was an independent risk factor for atherothrombotic cerebral infarction onset in females^21^. Our results also imply a possible effect of haplogroup A on stroke outcomes, which cannot be justify because of the multiple comparisons problem.

There are several limitations which should be addressed before interpreting the results. Pre-treatments with drugs, such as antiplatelet, angiotensin-converting-enzyme inhibitor and calcium channel blockers, may influence stroke prognosis^17^, however, we did not design to collect data of these factors as potential confounders. Due to the limited sample size, we cannot identify all possible mtDNA haplogroups reported in others studies. However, we included major haplogroups reported in Chinese population. This study is underpowered to detect precise risk loci for stroke outcomes, which means we cannot precisely locate the target gene. Therefore, this study cannot provide evidences from functional analysis. Furthermore, having fewer patients in the "worsened" group compared to the other two groups may jeopardize the statistical power. We did not test the impacts of mtDNA haplogroups on the evolvement of ischemic penumbra and infarct volume after stroke, but this kind of studies may be important for further illuminating the link between mtDNA haplogroups and stroke outcomes. Nuclear genetic background may also influence stroke outcomes, which emphasize the importance of studies that involve both mtDNA and nuclear DNA.

In conclusion, mitochondrial DNA haplogroup N9 was an independent protective factor for neurological worsening in patients with moderate to severe ischemic stroke. The results supported that mtDNA variants play a role in post-stroke neurological recovery, thus providing evidences for pharmacological intervention in mitochondrial function. Further studies to identify precise risk loci and clinical assessments, including ischemic penumbra, infarct volume and post-stroke neurological outcome, are warranted.

## Methods

### Study Population and Patient Selection

The study was approved by the Institutional Review Board of Jinling Hospital (Nanjing, China). A hospital based cohort of stroke patients was recruited between December 2009 and October 2013. In the recruitment, Han Chinese patients with first-ever ischemic stroke, and aged ≥ 18 years were included. Considering that the effects of mtDNA variants on neurological function may not be obvious in both minor and very severe stroke, we limited patients to those with a National Institutes of Health Stroke Scale (NIHSS) score between 5 and 20. Patients with concomitant diseases which may influence neurological outcomes, such as myopathy, respiratory failure and heart failure were excluded. Stroke severity and neurological function were measured with NIHSS. Clinical outcome was measured with modified Rankin Scale (mRS) on the 14th day of stroke onset. Signed informed consent was received from all participants. The methods were carried out in accordance with the approved guidelines.

### Short-term Outcomes of Ischemic Stroke

The baseline NIHSS was evaluated within 24 hours of stroke onset. The follow-up NIHSS was assessed on the 14th day. If patients did not survive to the 14th day, the follow-up NIHSS scores were assumed as 42 points. Based on the change of NIHSS scores between baseline and follow-up, the outcomes of neurological functions were classified as improved (NIHSS score decreased by ≥ 2 points), stabilized (NIHSS score unchanged or deceased by 1 point) and worsened (NIHSS score increased by ≥ 1 point)^34^,^35^.

### Selection of SNPs for determining mtDNA haplogroups

Candidate single nucleotide polymorphisms (SNPs) were selected based on previously reported mtDNA phylogeny in East Asian populations^36^,^37^. Latest SNPs information of haplogroups was derived from PhyloTree.org - mtDNA tree Build 16^38^, and all position numbers are relative to the revised Cambridge reference sequence (rCRS)^39^. With plentiful SNPs for determining mtDNA haplogroups, the two hypervariable segment including HVS-I (16024-16383) and HVS-II (57-372) in control region were selected. In the coding region, segment 10400-10915 was selected, because this segment includes m.10400 and m.10873 whose information is necessary for determining macro-haplogroup M and N. The other sequence information of this segment was also useful for haplogrouping in East Asian mtDNA phylogeny. As a result, segment 15901-16497 (including HVS-1), segment 42-385 (including HVS-II) and segment 10400-10915 were sequenced for each sample in this study.

### MtDNA Sequencing and haplogrouping

Venous blood was obtained from each patient, from which mtDNA was exacted using RelaxGene Blood DNA systems (TIANGEN Biotech Co., Ltd., Beijing). The Sanger method was performed to sequence the three segments of mtDNA. Primer3 (http://bioinfo.ut.ee/primer3-0.4.0/)^40^,^41^ was used to design polymerase chain reaction (PCR) primers (see Supplementary Table S5 online). MtDNA was sequenced using ABI3130XL DNA Sequencer and BigDye Terminator v3.1 Cycle Sequencing Kit (Applied Biosystems, USA). SNPs were detected with PolyPhred (http://droog.gs.washington.edu/polyphred/). Negative controls were included in each plate to ensure accuracy of the genotyping. About 5% samples were randomly selected to repeat the sequencing, and the two results reached a 100% concordance.

All samples were assigned into mitochondrial macro-haplogroup M (with m.10400C>T), N (with m.10873C>T) and L3’4 (without m.10400C>T and m.10873C>T). Variants information in control region was used for further haplogrouping with MitoTool 1.1.2 released (PhyloTree Build 16 database)^42^,^43^.

### Statistical Analysis

Statistical analysis was performed using SPSS Statistics Version 22.0 (Armonk, NY: IBM Corp.) and R v3.1.1 (http://www.R-project.org/)^44^. Normal distributions of continuous data were tested using Shapiro-Wilk test. As all continuous data in this study did not meet the normality assumption, non-parametric tests were used instead. Continuous and ordinal data were compared with Mann-Whitney or Kruskal-Wallis tests. Frequency distributions of nominal data are compared using Fisher's exact test. The correlation mRS scores and outcomes of neurological function based on the NIHSS score change were analyzed with Spearman's rank correlation test. Ordinal regression analysis (proportional odds model) was performed to evaluate the associations between mtDNA haplogroups and outcomes of neurological functions. Multivariate logistic regression analysis was performed to evaluate the associations between mtDNA haplogroups and short-term clinical outcomes. The Bonferroni method was used for correcting the *P* values involved multiple comparisons. Statistical significance was examined by two-sided test, and the significance threshold was set at 0.05.

## Author Contributions

B.C. and G.X. conceived and designed the experiments, and wrote the draft of the manuscript; B.C. and Z.Z. undertook the statistical analyses; B.C., K.L., W.F., M.D. and J.D. collected data; B.C., Y.Z., X.X., L.C., W.B., S.Z. and H.Z. performed laboratory experiments; Q.D., Z.Z. and S.Z. gave critical comments on the draft and contributed to the manuscript writing; W.Z., M.M. and W.L. conducted clinical assessments during hospitalization and follow-up; G.X. and X.L. reviewed clinical assessments in this study and supervised this study.

## Additional Information

**How to cite this article**: Cai, B. *et al* . Mitochondrial DNA haplogroups and short-term neurological outcomes of ischemic stroke. *Sci. Rep.* **5**, 9864; doi: 10.1038/srep09864 (2015).

## Supplementary Material

Supplementary Information Supplementary Information

## Figures and Tables

**Table 1:** Demographic and clinical characteristics by outcomes of neurological functions.

Characteristics	All	Outcomes of Neurological Functions			*P* value
		Improved	Stabilized	Worsened	
	n = 303	n = 162	n = 103	n = 38	
Age, years	60.0 (50.0-67.0)	59.0 (48.0-68.0)	61.0 (51.0-67.0)	59.5 (54.5-68.0)	0.785
Sex, male (%)	223 (73.6)	125 (77.2)	75 (72.8)	23 (60.5)	0.065
Hypertension (%)	186 (61.4)	81 (50.0)	74 (71.8)	31 (81.6)	**<0.001**
Diabetes mellitus (%)	83 (27.4)	33 (20.4)	37 (35.9)	13 (34.2)	**0.006**
Atrial fibrillation (%)	19 (6.3)	13 (8.0)	3 (2.9)	3 (7.9)	0.316
Smoking (%)	114 (37.6)	63 (38.9)	37 (35.9)	14 (36.8)	0.659
Alcohol drinking (%)	88 (29.0)	49 (30.2)	34 (33.0)	5 (36.8)	0.271
TG, mmol / L	1.40 (1.01-1.85)	1.42 (0.99-1.91)	1.41 (1.12-1.92)	1.37 (1.12-1.61)	0.538
Chol, mmol / L	4.50 (3.69-5.10)	4.40 (3.80-5.07)	4.51 (3.60-5.10)	4.59 (3.53-5.03)	0.847
HDL, mmol / L	1.06 (0.88-1.22)	1.06 (0.88-1.21)	1.05 (0.88-1.19)	1.08 (0.89-1.34)	0.689
LDL, mmol / L	2.68 (2.13-3.30)	2.66 (2.20-3.25)	2.63 (2.00-3.37)	2.83 (2.10-3.23)	0.864
TOAST subtypes					0.403
LAA	207 (68.3)	103 (63.6)	78 (75.7)	26 (68.4)	
CES	30 (9.9)	18 (11.1)	7 (6.8)	5 (13.2)	
SVS	38 (12.5)	22 (13.6)	13 (12.6)	3 (7.9)	
UND	28 (9.2)	19 (11.7)	5 (4.9)	4 (10.5)	
Treatments					
IV thrombolysis (%)	10 (3.3)	5 (3.1)	1 (1.0)	4 (10.5)	0.327
Antihypertensive (%)	159 (52.5)	77 (47.5)	55 (53.4)	27 (71.1)	**0.023**
Glucose-lowering (%)	86 (28.4)	37 (22.8)	35 (34.0)	14 (36.8)	**0.022**
Antiplatelet (%)	295 (97.4)	156 (96.3)	102 (99.0)	37 (97.4)	0.298
Anticoagulant (%)	53 (17.5)	27 (16.7)	19 (18.4)	7 (18.4)	0.698
Statin (%)	293 (96.7)	153 (94.4)	102 (99.0)	38 (100)	**0.019**
Clinical assessment					
Baseline NIHSS	8 (6-11)	8 (6-11)	7 (6-12)	7 (5-9)	0.081
mRS	3 (2-4)	3 (1.75-4)	4 (3-4)	4 (3.75-5)	**<0.001**

Data are presented as number of individuals (%) or median (interquartile range). TG = triglyceride; Chol = cholesterol; HDL = high density lipoprotein; LDL = low density lipoprotein; TOAST = Trial of Org 10172 in Acute Stroke Treatment; LAA = large-artery atherosclerosis; CES = cardiac embolism stroke; SVS = small-vessel stroke; UND = other determined and undetermined causes; IV = intravenous; NIHSS = National Institutes of Health Stroke Scale; mRS = modified Rankin Scale.

**Table 2:** Results of haplogroups associated with outcomes of neurological functions in the ordinal regression model (adjusted for age, hypertension, diabetes mellitus, TOAST subtypes, antihypertensive, glucose-lowering and statin treatment).

Haplogroup	All	Outcomes of Neurological Functions			OR (95% CI)	*P* value
		Improved	Stabilized	Worsened		
A	20	12	4	4	0.82 (0.48-1.40)	0.468
B	41	19	15	7	1.34 (0.92-1.96)	0.126
D	64	35	25	4	0.91 (0.66-1.26)	0.578
G	16	8	5	3	1.18 (0.65-2.12)	0.588
M7	25	14	6	5	1.24 (0.76-2.02)	0.393
M8	27	13	13	1	0.90 (0.57-1.43)	0.666
N9	19	15	4	0	0.39 (0.20-0.75)	**0.005**
R9	40	16	17	7	1.31 (0.90-1.90)	0.155
Other	51	30	14	7	-	-
Total	303	162	103	38	-	-

OR = odds ratio; CI = confidence interval.
