# Supplementary material for: Mitochondrial DNA haplogroups and short-term neurological outcomes of ischemic stroke
Source: Sci Rep. 2015 May 20;5:9864. doi: 10.1038/srep09864 (PMC4438613; doi:10.1038/srep09864)
Supplement: Supplementary Information [file srep09864-s1.pdf]

Supplementary Information

Mitochondrial DNA haplogroups and short-term neurological outcomes of Ischemic Stroke

Biyang Cai[1*], Zhizhong Zhang[1*], Keting Liu[2], Wenping Fan[1], Yumeng Zhang[1], Xia Xie[2], Minhui Dai[1], Liping Cao[3], Wen Bai[4], Juan Du[5], Qiliang Dai[1], Shuyu Zhou[1], Hao Zhang[1, 6], Wusheng Zhu[1], Minmin Ma[1], Wenhua Liu[1], Xinfeng Liu[1], Gelin Xu[1]

[1] Department of Neurology, Jinling Hospital, Medical School of Nanjing University, Nanjing 210002, Jiangsu, China

[2] Department of Neurology, Jinling Hospital, Southern Medical University, Nanjing 210002, Jiangsu, China

[3] Department of Neurology, The First People's Hospital of Changzhou, Changzhou 213003, Jiangsu, China

[4] Department of Neurology, Yantai Yuhuangding Hospital, Yantai 264000, Shandong, China

[5] Department of Neurology, Jinling Hospital, Second Military Medical University, Nanjing 210002, Jiangsu, China

[6] Department of Neurology, The First People's Hospital of Hangzhou, Nanjing Medical University, Hangzhou 310006, Zhejiang, China

*These authors contributed equally to this work.

Supplementary Table S1. Frequencies of mitochondrial DNA haplogroups.

Supplementary Table S2. Demographic and clinical characteristics for mtDNA haplogroups.

Supplementary Table S3. Demographic and clinical characteristics by short-term clinical outcomes.

Supplementary Table S4. Result of haplogroup associated with short-term clinical outcomes in the multivariate logistic regression model (adjusted for age, baseline NIHSS score and TOAST subtypes).

Supplementary Table S5. Polymerase chain reaction primers designed for mitochondrial DNA sequencing.

Supplementary Figure S1. Proportions of mRS (modified Rankin Scale) scores for mitochondrial DNA haplogroups.

Supplementary Table S1. Frequencies of mitochondrial DNA haplogroups.

Common ancestor haplogroup	Haplogroup	Frequency	Percent
A	A	20	6.6%
B	B4	27	8.9%
	B5	14	4.6%
D	D	1	0.3%
	D4	4	1.3%
	D4a	14	4.6%
	D4b	7	2.3%
	D4d	1	0.3%
	D4e	3	1.0%
	D4g	6	2.0%
	D4h	2	0.7%
	D4k	1	0.3%
	D4q	2	0.7%
	D4t	3	1.0%
	D5	17	5.6%
	D5a	2	0.7%
	D5b	1	0.3%
G	G1	2	0.7%
	G2	8	2.6%
	G3	6	2.0%
M7	M7b	17	5.6%
	M7c	8	2.6%
M8	M8a	6	2.0%
	C	14	4.6%
	Z	7	2.3%

Supplementary Table S1. Frequencies of mitochondrial DNA haplogroups.

Common ancestor haplogroup	Haplogroup	Frequency	Percent
N9	N9a	16	5.3%
	Y1	3	1.0%
R9	R9b	2	0.7%
	F1	13	4.3%
	F2	17	5.6%
	F3	5	1.7%
	F4	3	1.0%
	M	25	8.3%
	M9a	5	1.7%
	M9b	1	0.3%
	M10a	4	1.3%
	M13a	1	0.3%
	M74a	1	0.3%
Other	N	2	0.7%
	R	3	1.0%
	R11'B	4	1.3%
	H66a	1	0.3%
	N10a	1	0.3%
	T1a1'	1	0.3%
	L3'4	2	0.7%
Total		303	100.0%

Supplementary Table S2. Demographic and clinical characteristics for mtDNA haplogroups.

Characteristic	Haplogroup									All	*P* value
	A	B	D	G	M7	M8	N9	R9	Other		
n (%)	20 (6.6)	41 (13.5)	64 (21.1)	16 (5.3)	25 (8.3)	27 (8.9)	19 (6.3)	40 (13.2)	51 (16.8)	303 (100)	
Age, years	65.5 (54.3-70.8)	62.0 (55.5-69.0)	58.0 (50.3-69.3)	56.0 (48.8-67.0)	64.0 (47.5-66.0)	61.0 (50.0-68.0)	53.0 (47.0-64.0)	51.0 (60.0-67.3)	48.0 (59.5-71.3)	60.0 (50.0-67.0)	0.525
Sex, male (%)	10 (50.0)	29 (70.7)	49 (76.6)	13 (81.3)	18 (72.0)	16 (59.3)	17 (89.5)	31 (77.5)	40 (78.4)	223 (73.6)	0.138
Hypertension (%)	17 (85.0)	24 (58.5)	35 (54.7)	7 (43.8)	15 (60.0)	18 (66.7)	11 (57.9)	28 (70.0)	31 (60.8)	186 (61.4)	0.260
Diabetes mellitus (%)	5 (25.0)	8 (19.5)	18 (28.1)	7 (43.8)	6 (24.0)	7 (25.9)	4 (21.1)	15 (37.5)	13 (25.5)	83 (27.4)	0.653
Atrial fibrillation (%)	1 (5.0)	3 (7.3)	5 (7.8)	1 (6.3)	1 (4.0)	0 (0)	2 (10.5)	2 (5.0)	4 (7.8)	19 (6.3)	0.912
Smoking (%)	4 (20.0)	18 (43.9)	24 (37.5)	5 (31.3)	7 (28.0)	10 (37.0)	8 (42.1)	12 (30.0)	26 (51.0)	114 (37.6)	0.305
Alcohol drinking (%)	3 (15.0)	14 (34.1)	21 (32.8)	4 (25.0)	6 (24.0)	8 (29.6)	4 (21.1)	9 (22.5)	19 (37.3)	88 (29.0)	0.638
TG, mmol / L	1.48 (1.02-2.54)	1.48 (1.34-1.91)	1.27 (1.00-1.90)	1.62 (1.31-1.92)	1.23 (0.99-1.63)	1.47 (1.00-2.06)	1.49 (0.89-2.14)	1.00 (1.43-1.87)	0.96 (1.27-1.71)	1.40 (1.01-1.85)	0.458
Chol, mmol / L	4.60 (3.82-5.24)	4.40 (3.90-4.85)	4.40 (3.50-5.00)	4.90 (3.50-6.58)	4.60 (3.70-5.00)	4.15 (3.60-5.15)	4.80 (3.89-5.50)	3.89 (4.80-5.10)	3.63 (4.40-5.02)	4.50 (3.69-5.10)	0.747
HDL, mmol / L	1.16 (0.91-1.32)	1.04 (0.89-1.26)	1.00 (0.79-1.19)	1.12 (0.88-1.33)	1.08 (0.89-1.28)	1.03 (0.91-1.08)	1.14 (0.88-1.23)	0.87 (1.11-1.21)	0.83 (1.50-1.25)	1.06 (0.88-1.22)	0.846
LDL, mmol / L	2.66 (2.02-3.21)	2.60 (2.17-3.17)	2.57 (2.00-3.58)	3.16 (1.86-4.20)	2.75 (2.23-3.55)	2.46 (2.03-3.30)	2.72 (1.92-3.35)	2.15 (2.91-3.32)	2.30 (2.53-3.22)	2.68 (2.13-3.30)	0.903
TOAST subtypes											0.466*
LAA	15 (75.0)	26 (63.4)	39 (60.9)	14 (87.5)	14 (56.0)	19 (70.4)	13 (68.4)	31 (77.5)	36 (70.6)	207 (68.3)	
CES	4 (20.0)	4 (9.8)	7 (10.9)	1 (6.3)	1 (4.0)	1 (3.7)	3 (15.8)	4 (10.0)	5 (9.8)	30 (9.9)	
SVS	1 (5.0)	5 (12.2)	11 (17.2)	1 (6.3)	5 (20.0)	5 (18.5)	2 (10.5)	4 (10.0)	4 (7.8)	38 (12.5)	
UND	0 (0)	6 (14.6)	7 (10.9)	0 (0)	5 (20.0)	2 (7.4)	1 (5.3)	1 (2.5)	6 (11.8)	28 (9.2)	
Clinical assessments											
Baseline NIHSS	6.5 (5-10.75)	9 (6-12)	8 (6-11)	10 (7.25-12.75)	7 (6-11)	7 (5-10)	10 (6-13)	5 (6-8.25)	6 (9.5-13.5)	8 (6-11)	**0.049**
Follow-up NIHSS	5.5 (2.25-9.5)	7 (4.5-11)	6 (4-9)	8 (7-10.75)	7 (4-10.5)	5 (3-9)	8 (5-12)	6 (4.25-8)	8 (4-11)	6 (4-10)	0.227
14-day mRS	3 (1.25-4)	3 (3-4)	3 (2-4)	4 (3-4)	3 (1-4)	3 (2-4)	3 (2-4)	2 (3-4)	2.75 (3-4)	3 (2-4)	0.110

Data are presented as number of individuals (%) or median (interquartile range). TG = triglyceride; Chol = cholesterol; HDL = high density lipoprotein; LDL = low density lipoprotein; TOAST = Trial of Org 10172 in Acute Stroke Treatment; LAA = large-artery atherosclerosis; CES = cardiac embolism stroke; SVS = small-vessel stroke; UND = other determined and undetermined causes; NIHSS = National Institutes of Health

Stroke Scale; mRS = modified Rankin Scale.

* The Monte Carlo P value (Chi-squared test) base on 10,000 samples was calculated instead, because of the limited computer capacity.

Supplementary Table S3. Demographic and clinical characteristics by short-term clinical outcomes.

Characteristics	All	Short-term clinical outcomes		*P* value
		Good	Poor	
	n = 303	n = 102	n = 201	
Age, years	60.0 (50.0-67.0)	63.0 (53.0-68.0)	59.0 (48.5-67.0)	0.128
Sex, male (%)	223 (73.6)	79 (77.5)	144 (71.6)	0.335
Hypertension (%)	186 (61.4)	58 (56.9)	128 (63.7)	0.263
Diabetes mellitus (%)	83 (27.4)	25 (24.5)	58 (28.9)	0.496
Atrial fibrillation (%)	19 (6.3)	10 (9.8)	9 (4.5)	0.082
Smoking (%)	114 (37.6)	34 (33.3)	80 (39.8)	0.316
Drinking (%)	88 (29.0)	28 (27.5)	60 (29.9)	0.690
TG, mmol / L	1.40 (1.01-1.85)	1.24 (1.01-1.86)	1.47 (1.03-1.85)	0.298
Chol, mmol / L	4.50 (3.69-5.10)	4.40 (3.70-5.00)	4.53 (3.63-5.10)	0.867
HDL, mmol / L	1.06 (0.88-1.22)	1.07 (0.88-1.20)	1.05 (0.88-1.25)	0.945
LDL, mmol / L	2.68 (2.13-3.30)	2.60 (2.10-3.28)	2.69 (2.20-3.33)	0.448
Baseline NIHSS	8 (6-11)	6 (5-7)	10 (7-13)	**<0.001**
TOAST subtypes				**0.007**
LAA	207 (68.3)	59 (57.8)	148 (73.6)	
CES	30 (9.9)	15 (14.7)	15 (7.5)	
SVS	38 (12.5)	20 (19.6)	18 (9.0)	
UND	28 (9.2)	8 (7.8)	20 (10.0)	
Treatments				
IV thrombolysis (%)	10 (3.3)	3 (2.9)	7 (3.5)	0.803
Antihypertensive (%)	159 (52.5)	54 (52.9)	105 (52.2)	1.000
Glucose-lowering (%)	86 (28.4)	26 (25.5)	60 (29.9)	0.501
Antiplatelet (%)	295 (97.4)	99 (97.1)	196 (97.5)	1.000
Anticoagulant (%)	53 (17.5)	13 (12.7)	40 (19.9)	0.150
Statin (%)	293 (96.7)	97 (95.1)	196 (97.5)	0.313

Data are presented as number of individuals (%) or median (interquartile range). TG =

triglyceride; Chol = cholesterol; HDL = high density lipoprotein; LDL = low density lipoprotein; TOAST = Trial of Org 10172 in Acute Stroke Treatment; LAA = large-artery atherosclerosis; CES = cardiac embolism stroke; SVS = small-vessel stroke; UND = other determined and undetermined causes; IV = intravenous; NIHSS = National Institutes of Health Stroke Scale.

Supplementary Table S4. Result of haplogroup associated with short-term clinical outcomes in the multivariate logistic regression model (adjusted for age, baseline NIHSS score and TOAST subtypes).

Haplogroup	All	Short-term clinical outcome		OR (95% CI)	*P* value
		Good	Poor		
A	20	7	13	1.27 (0.57-5.21)	0.338
B	41	8	33	2.32 (0.92-5.87)	0.076
D	64	28	36	0.54 (0.26-1.08)	0.081
G	16	3	13	1.33 (0.31-5.74)	0.700
M7	25	9	16	0.80 (0.29-2.24)	0.678
M8	27	11	16	0.76 (0.29-1.97)	0.573
N9	19	6	13	0.91 (0.24-3.41)	0.887
R9	40	18	22	0.83 (0.37-1.85)	0.651
Other	51	12	39	-	-
Total	303	102	201	-	-

OR = odds ratio; CI = confidence interval.

Supplementary Table S5. Polymerase chain reaction primers designed for mitochondrial DNA sequencing.

Segment	Forward and reverse	Primer sequence	Product size (bp)
1	Forward	TCAAATGGGCCTGTCCTTGT	675
	Reverse	AGGGGAACGTGTGGGCTATT	
2	Forward	ACATCACGATGGATCACAGGTC	434
	Reverse	AAAGTGCATACCGCCAAAAGA	
3	Forward	GCCCTAAGTCTGGCCTATGAG	587
	Reverse	GGGGTCGGAGGAAAAGGT	

bp = base pair.

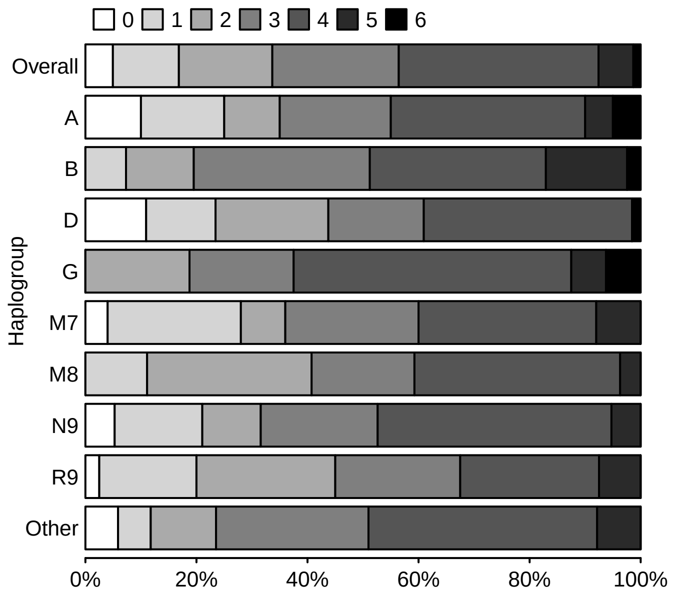

Supplementary Figure S1. Proportions of mRS (modified Rankin Scale) scores for mitochondrial DNA haplogroups.
